# Big Data, Biomedical Research, and Ethics Review: New Challenges for IRBs

**DOI:** 10.1002/eahr.500065

**Published:** 2020-09-16

**Authors:** Agata Ferretti, Marcello Ienca, Samia Hurst, Effy Vayena

**Affiliations:** ^1^ PhD candidate in Bioethics at the Health Ethics and Policy Lab, Swiss Federal Institute of Technology in Zurich; ^2^ Senior researcher at the Health Ethics and Policy Lab, Swiss Federal Institute of Technology in Zurich; ^3^ Professor of medical ethics and director of the Institute for Ethics, History, and the Humanities & Department of Community Health and Medicine, University of Geneva, Switzerland; ^4^ Professor of bioethics and director of the Health Ethics and Policy Lab, Swiss Federal Institute of Technology in Zurich

**Keywords:** big data, big data research, biomedical research, research ethics, institutional review board (IRB)

## Abstract

The increased use of big data in the medical field has shifted the way in which biomedical research is designed and carried out. The novelty of techniques and methods brought by big data research brings new challenges to institutional review boards (IRBs). Yet it is unclear if IRBs should be the responsible oversight bodies for big data research and, if so, which criteria they should use. A large but heterogenous set of ethics guidelines and normative responses have emerged to address these issues. In this study, we conducted a scoping review of soft‐law documents and guidelines with the aim of assessing ongoing normative efforts that are proliferating in this domain. We also synthesize a set of recurrent guidelines that could work as a baseline to create a harmonized process for big data research ethics.

**T**raditionally, human subjects research in the biomedical field engages healthy and sick people as research participants in order to test certain hypotheses about health and disease according to a well‐defined study design. The study designs, such as randomized controlled trials and cohort studies, are carefully reviewed by institutional review boards (IRBs)—also known as *ethical review committees* (*ERCs*) in some countries. IRBs are committed to protecting the rights and welfare of human subjects recruited to participate in biomedical or behavioral research (including social science research).^1^ This approach has long been the standard for biomedical research.

Recently, however, biomedical research has begun to pursue opportunities afforded by big data. Big data research relies on large‐scale databases, multiplication of data sources, advanced storage capacity, and novel computational tools that allow for high‐velocity data analytics.^2^ In the biomedical domain, big data trends are enabled by and allow for advances in areas such as whole genome sequencing, brain imaging, mobile health, and digital phenotyping.^3^ Today, a large portion of health‐related research relies on big data. Big data also enables researchers to draw health insights from data sources that are not strictly medical—data from wearable trackers, social media, and Internet searches, for example.^4^ Big data research opens new prospects to accelerate health‐related research and potentially elicit breakthroughs that will benefit patients.^5^

Big data has been observed to shift the way biomedical researchers design and carry out their studies.^6^ This research departs from the traditional research model because it is largely exploratory rather than hypothesis driven. Health‐related big data research is based on the acquisition of large amounts of data from multiple and often heterogeneous sources, which are subsequently combined and mined using powerful data analytics tools. This reverse‐engineered approach to health‐related research allows researchers to extract features and valuable insights from large datasets, without being able to anticipate exactly what the data analysis will find.

The methodological novelty of big data research models brings new challenges and questions to IRBs, including whether they are the bodies responsible for assessing these projects, and if they are, what criteria they should use to evaluate them. Given current technologies, analytic methods, and regulations, IRBs cannot take their traditional review frameworks as given. This is because big data research models might not fit within the traditional national review policies for the protection of human subjects (for example, the Common Rule in the United States and the Human Research Act in Switzerland) and the principles stated in guidelines documents such as the Helsinki Declaration of the World Medical Association and the U.S. National Commission's *Belmont Report*. It was observed that the definition of “human subjects” in the Common Rule might not cover big data projects involving the processing of deidentified data.^7^ The Common Rule's scope, in fact, is limited to the acquisition and processing of “identifiable private information.” As a consequence, privately held, publicly available datasets such as Twitter data might be considered exempt from IRB oversight, even though it is possible to reidentify those data sources by matching them with ancillary information.^8^ Similarly, the European Union's research ethics legislation,^9^ as well as the Swiss Human Research Act,^10^ might not apply to research that involves anonymized data or secondary use of data for which a broad consent and an ERC approval was obtained. For instance, in Denmark, researchers can reuse genomic data previously extracted from a donated tissue sample of the National Biobank Registry for a new project without seeking ERC approval.^11^

Health‐related big data research also challenges IRBs in referring to existing safeguards for ethics research such as informed consent, privacy and confidentiality, and minimal risk.^12^ The reason for that stems from a threefold consideration.

First, individuals whose data are used in research (hereafter data subjects) are often not sufficiently informed concerning the use of their data. Particularly, researchers might not be able to adequately inform data subjects when collecting their data stored in large repositories or when mining the data in the context of secondary data uses. In a more pragmatic sense, informed consent might be hard to obtain in big data studies due to the high number of data subjects involved. This is particularly true when consent is sought retrospectively. In cases where research is conducted on large‐scale repositories, it might be hardly feasible to recontact all data subjects and inform them that the purpose of data processing has changed from the original consent agreement stipulated at the time when the repository was created.

Second, breaches in data privacy and confidentiality represent a major source of risk for health research using big data. The reason for that stems from the informational richness of large research data repositories, which makes them a primary target for actors outside the research domain. Insurers, marketing companies, and the government might require access to these data. Furthermore, health‐related data repositories have often been exposed to illicit use by malevolent actors. Although these repositories are usually composed of data that do not contain personal identifiers (deidentified data), research has shown that both pseudonymized data (with which artificial identifiers are used so data subjects can be reidentified) and anonymized data (from which actual and artificial identifiers are excluded in an effort to make reidentification impossible) could be matched with publicly available information or auxiliary data to allow the reidentification of a subject.^13^ This reidentification risk is particularly problematic for health research, as health data constitute a highly sensitive data source.

Finally, correlations arising from health‐related big data analytics can be abused by various actors for unethical purposes such as discriminating against applicants to health insurance services or jobs based on health risk indicators. These indicators include, among others, risk factors associated with genetic variants, neuroimaging biomarkers of addiction or antisocial behavior, and molecular biomarkers of chronic illness. This risk of discrimination also applies to not strictly medical data such as online behavioral information. For example, a recent study has used a big data approach to predict people's sexual orientation from their online behavior.^14^ This poses a risk to many people, especially in countries where nonheterosexual behavior is prohibited by law. Although the research involved the processing of seemingly innocuous data points, the findings suggest that the risk of reidentification is potentially greater than minimal risk.

Many ethical issues remain to be solved. These include whether and when big data projects using deidentified data from public databases should require IRB approval, what counts as “public data,” what constitutes “minimal‐risk” in data‐driven projects, and which novel ethical safeguards, if any, are required to ensure ethical big data research.

To reduce this uncertainty, various stakeholders have issued nonbinding guidelines. The scientific community has developed best‐practice guidelines and educational activities aimed at sensitizing researchers about the ethical promises and challenges of big data research. In parallel, a growing number of professional organizations are restructuring their codes of conduct and providing research ethics training to data and computer scientists. Members of the scientific community, such as the editors of the journal *Nature*, have encouraged policy‐makers to “further support such efforts… and make them better known to researchers” and have proclaimed that “all researchers have a duty to consider the ethics of their work beyond the strict limits of law or today's regulations.”^15^

Nevertheless, the proliferation of many independent responses around big data research ethics has generated uncertainty among IRBs. This fragmented landscape of responses increases the confusion and leaves IRBs with unclear normative guidance about how to tackle big data ethics issues. We monitored and evaluated these efforts to bring clarity about the plurality of perspectives emerging in this domain. To accomplish our purpose, we have conducted a scoping review of the soft‐law documents and guidelines^16^ concerning the ethics of health‐related big data research. While previous reviews have screened the scholarly literature on this topic,^17^ and opinion articles have discussed their implications for IRBs,^18^ no other study, to our knowledge, has provided a comprehensive assessment of the emerging body of guidelines on this topic. Research best practices, recommendations, codes of conduct, and other guidance documents—especially those commissioned by funding agencies, professional associations, academic societies, nongovernmental organizations (NGOs), think tanks, and private companies—are typically not formally published in peer‐reviewed academic journals but, rather, released in commissioned technical reports, white papers, and similar documents. Because these types of documents are usually not indexed in academic archives and databases, reviewing this gray literature^19^ is critical to retrieve and assess this body of information. We believe that IRBs will benefit from our research, as we provide a comprehensive set of recommendations that could represent the starting point for IRBs in revising and harmonizing their ethics research processes.

## APPROACH TO IDENTIFYING DOCUMENTS FOR REVIEW

**I**n February 2019, we conducted an online search of the gray literature addressing the ethical implications of health‐related big data research. Gray literature

**A few documents addressed the issue of whether IRBs should be the oversight body accountable for big data research at all. Any development of an ethical framework for big data research, however, cannot disregard the active involvement of IRBs in decision‐making**.

is defined as “literature that is not formally published in sources such as books or journal articles.”^20^ This definition includes nonconventional material such as reports, technical specifications and standards, technical and commercial documentation, official documents,^21^ and “that which is produced on all levels of government, academics, business and industry in print and electronic formats, but which is not controlled by commercial publishing interests and where publishing is not the primary activity of the organisation.”^22^ Gray literature reviews have been observed to have the threefold advantage of providing information of process and implementation, both of which can be missing from scientific papers;^23^ reducing the typical publication lag of peer‐reviewed articles, hence ensuring more efficient responses;^24^ and validating the results of research‐based literature searches.^25^

Following previous studies,^26^ we used a multistage screening process involving both inductive screening via search engine and deductive identification of relevant agencies (for example, national, international, and intergovernmental organizations), and subsequently we screened their websites and online collections. A total of 49 documents were included in our analysis (see appendix 1, available online, along with all the figures and the appendices; see “Supporting Information” at the end of this article). In the literature retrieval phase, we searched the Google engine in nonpersonalized mode using multiple combinations of the following keywords: “big data,” “data science,” “digital data,” “medical,” “healthcare,” “clinical,” “policy,” “ethics,” “governance,” “ethics committee,” “IRB,” and “ethics review board.” Combinations of keywords were reiterated until saturation was achieved. In the screening phase, we selected, on the one hand, soft‐law documents issued by national and international agencies (highlighted in blue in appendix 1) and, on the other hand, nonlegal guidelines providing best practices and recommendations, disseminated by NGOs, professional organizations, research bodies, private companies, think tanks, and other actors (highlighted in yellow in appendix 1).^27^ We included only official documents representative of and issued by collective entities. Documents such as personal blogs, written and issued by individual authors offering their personal views, were not included. In the filtering phase, we excluded from the analysis all documents that did not meet our content‐based inclusion criteria (see appendix 2). The documents were collected independently by two coauthors who compared their results and resolved interpretative discrepancies upon discussion. Documents written in English, Italian, French, Greek, and German (languages spoken by research team members) were included in the analysis. We then conducted a descriptive numerical summary and a thematic analysis. The descriptive numerical summary consisted of calculating the frequencies of the total number of articles included, the distribution of documents by the documents’ issuers and the targeted stakeholder group, and the prevalence of documents with a particular health‐related or IRB focus. In the latter analysis, two researchers inductively identified recurrent themes^28^ with software assistance. The two researchers coded the themes using the NVivo software for qualitative data analysis (version 12 for Mac) considering three macro areas: general ethical issues, normative ethical recommendations, and specific recommendations for IRBs. Within these areas, we grouped and merged our coding in themes and macrothemes.^29^ Disagreement about where to allocate codes that did not seem to follow in any existing theme was resolved among coauthors through internal consultation.

## LITERATURE REVIEW RESULTS

**M**ost documents were issued by national and governmental institutions (28%), followed by professional organizations (22%) and NGOs (20%). Fewer documents (no more than five) were issued by private companies, international institutions, research institutions, and think‐tank platforms. The geographical provenance of the issuers (41% from North America, 35% from Europe, 20% being international (“international” meaning that the issuer of that document was not located in a specific country; rather, it was a multicountry agency), and 4% from Oceania) showed a higher representation of highly industrialized Western countries and a relative underrepresentation of low‐ and middle‐income countries from the global south. The majority of the documents (35%) targeted readers from various stakeholder groups (for instance, government institutions, researchers, professionals, industry associations, consumer advocates, and the general public), with a quarter specifically targeting governmental regulations and a fifth addressing professional groups (e.g., the professional organization of statisticians) (see figure 1).

As to the content of the documents, 55% had a prominent focus on health‐related big data, with the remaining 45% having a broader focus on big data research, including health‐research themes. Additional analysis revealed that only 16 documents (33%) were addressed explicitly to IRBs or ERCs and provided ad hoc recommendations for the review of big data projects. The remaining documents provided general recommendations concerning the general ELSI (ethical, legal, and social implications) of big data research, but they do not address IRBs directly.

Our inductive thematic analysis identified a number of mutually interconnected ethical themes (see figure 2). The notion of privacy was by far the most prevalent; it was mentioned in all documents and discussed in depth in three quarters of them. A highly recurrent issue concerned how to balance data providers’ privacy while enabling the progress of research using big data techniques. While the importance of preserving privacy was widely recognized across the literature we reviewed, some documents raised the problem of harmonizing privacy regulations: given that privacy regulations differ across different jurisdictions, it might not, documents observed, be straightforward for researchers and IRBs to determine how to ensure privacy in cross‐national big data research. Documents also addressed the problem of ensuring the semantic unambiguity of the privacy concept in spite of the blurred distinction between public and private data repositories in the digital ecosystem. For example, documents questioned whether health‐related big data projects that use data from public‐by‐default social media platforms such as Twitter should undergo similar privacy impact assessments and ethics review as conventional biomedical research does.

The second most common ethical theme was informed consent (discussed in 44% of documents), whose ethical sensitivity was primarily associated with the problem of obtaining retrospective consent from data subjects when conducting large‐scale big data studies. Documents questioned the practical feasibility of retrospectively contacting many hundreds of thousands of data subjects—such as during the emotional “contagion” study^30^—and discussed the ethical justification for seeking retrospective consent in the context of public health research conducted in the public interest (such as epidemic prevention). Data ethics issues associated with data management, such as data security (39%), data sharing (37%), and data transparency (40%), also composed a significant portion of the current ethical landscape. Issues of algorithmic bias, beneficence, the right to be forgotten, data ownership, and individual autonomy appeared less prevalent.

Contextual analysis of emergent themes identified the ethical, legal, social, or technical contexts within which each subtheme was discussed or a solution was proposed (see figure 2). Results showed that the documents discussed 41% of the themes in the context of normative ethical analysis and with respect to potential solutions relying on ethical guidelines, best practices, or conceptual clarification. A third of the themes (31%) were discussed with reference to their technical implications and solutions. For example, distributed‐ledger computing (or blockchain), encryption, and differential privacy were all mentioned as possible technical solutions to privacy risks in the big data domain.^31^ A smaller number of documents addressed the political and regulatory domain and proposed solutions in terms of novel legislation (18%) or social strategies (12%).

Analysis of thematic interrelations indicates a high degree of interconnectedness among (sub)themes and contexts. Although primarily discussed in the context of ethical analysis, privacy and informed consent issues were largely cross‐discussed in various contexts including the technical and legislative domains. Issues of data security and stakeholder collaboration were primarily discussed in connection with, respectively, technical solutions and social considerations. Data security was primarily presented as a problem requiring technical solutions (for example, enhanced encryption, immunization to abusive apps, and adherence to international standards such as the ISO/IEC 27001:2013^32^), whereas hard law was considered unavoidable to address issues of data ownership and to enforce the data subjects’ right to be forgotten. Among these regulatory solutions, the European Union's General Data Protection Regulation, implemented in May 2018, was perceived as setting a new standard for data subject rights. Our thematic analysis also retrieved substantive recommendations for the ethics review of big data research. These recommendations concerned a variety of thematic families (see figure 3) and presented notable divergences in terms of content and degree of specification.

From the perspective of substantive ethical content, the most recurrent recommendation was for IRBs to ensure that researchers are providing adequate information to data subjects as part of the process of obtaining their informed consent (n = 31, 63%). Further recommendations required data controllers and processers to protect the privacy of data subjects (n = 30, 61%) and to assure data transparency and the trustworthiness of data‐driven inferences (n = 26, 53%). A smaller portion of recommendations required oversight bodies and regulators to foster collaborative exchange among stakeholders about data uses (n = 23, 47%) and to provide new guidance to assess the benefits and harms of big data research (n = 21, 42%). Appendix 3 provides further information concerning the substantive ethical recommendations issued in different continents and by various stakeholders.

In terms of granularity or degree of specification, most recommendations made general normative statements about the importance of promoting certain ethical principles (such as privacy) without specifying who should promote those principles and how, or in which domain they should be promoted and for what reason. A smaller number of documents offered a list of specifications including domain‐specific and stakeholder‐specific sets of good practices. Among those, a small portion of documents provided explicit recommendations for IRBs or analogous ethics review oversight bodies. In‐depth thematic analysis of this subset of documents revealed four major procedural recommendations for IRBs (see appendix 4).

### Strengthen oversight function

IRB oversight should be required for big data research even when the research project does not involve the physical (offline) recruitment of human subjects and does not process personally identifiable data or entail direct foreseeable harms to data generators. Furthermore, the IRB's purview should be expanded to monitor the ethical soundness of big data projects throughout the whole data lifecycle. The IRB's control mechanisms should be able to audit each phase of the project, including research planning, data collection, analytics, and results dissemination. The IRBs must inspect if ethical safeguards are in place to protect individual and group‐level privacy, autonomy, safety, and the quality and transparency of data management. A few documents argued that IRBs should have the capacity to anticipate or prospectively identify if violations of data access rights might occur and to manage ethical risks associated with data disclosure—particularly when analytic techniques are in use that can allow data processors (or third parties) to reidentify individuals and reveal sensitive information. Given the expertise and independent role of IRBs, they were generally claimed to be well‐suited to guarantee an impartial and objective oversight of big data studies.

### Improve the review process

IRBs should improve their review process to account for the novel ethical challenges of big data studies. For example, documents noted that IRBs might need to reconsider how to review informed consent procedures in large‐scale data‐driven projects where traditional informed consent models might be unfeasible (especially in the case of secondary or tertiary data uses).^33^ Similarly, documents highlighted that balancing risks and benefits is increasingly complicated in the age of big data, as indirect and informational risks are harder to detect compared to the conventional physical risks of clinical research.^34^ Furthermore, risks such as personal data leakages across multiple data cycles are difficult to anticipate prior to data collection and during the ethics review phase. Documents highlighted the need “to create or expand accountable data ethics review processes”^35^ and/or develop a new ethical framework specific to big data research. Some documents also suggested the creation of new independent advisory boards whose function should be complementary to that of IRBs.^36^

### Build capacity and expand competencies

Documents proposed that IRB members should receive additional training in data science and expand their knowledge of the ethical challenges of big data research. Wherever necessary, IRBs should also consider diversifying their membership to include data scientists and data ethicists. Several documents noted that IRBs are often composed of stakeholders (such as lawyers, physicians, nurses, and laypeople) who rarely have received formal training in computer or data science. Building capacity and expanding competencies are critical to anticipate and promptly identify ethical challenges. Some documents hypothesized that doing so will thereby improve the credibility of IRBs from the point of view of researchers and will increase researchers’ willingness to undergo ethics review.^37^

### Engage with researchers

IRBs should engage more with researchers and involve them in the ethical evaluation of big data projects. The analyzed documents reported that to achieve this goal, IRBs should have an open dialogue with researchers, sensitize them about the importance of ethically aligned research, and develop facilitated channels for the ethics review of big data projects. At the same time, IRBs should get involved, together with academic ethicists, in the research ethics training of young data scientists. IRBs and researchers should establish a tight collaboration to identify, preempt, and manage ethical risks emerging in health‐related big data research. Overall, we recognized high heterogeneity in the way recommendations were carried out by different issuers (see appendix 4).

We note here several study limitations. In the phase of literature retrieval, three typical limitations of gray literature reviews applied: selection bias, the volatile structure of web content, and the documents’ heterogeneity. As our search string was written in English and our inclusion criteria included only articles written in any of the languages known by one or more of the researchers, articles written in any other language have not been included. While this limitation is inherent to any literature review, we believe we have minimized it by including articles written in five languages (English, French, German, Greek, and Italian). Another possible source of selection bias is that the Google search engine results are usually returned customized to a specific user and ranked following the number of hits a website received. To anticipate this problem, the search was performed independently by two researchers using separate terminals and IP addresses and in nonpersonalized mode. Google pages were screened until data saturation was reached and acknowledged by both researchers. To minimize subjective bias, the two researchers compared their results and, in cases of divergence, discussed them with motivations until agreement was reached.

The absence of an exhaustive repository of soft‐law and policy documents, together with the volatility of their web‐based content, might have also affected the review. On the one hand, relevant documents might have gone undetected due to the sensitivity of our search. On the other hand, retrieved documents might, in the future, be removed from the Internet. To minimize the first risk, two coauthors independently screened multiple Google results pages and then crosschecked their results. To address the latter, we retained the original documents in PDF format and created a private repository, which will be shared upon request.

Since we included in the analysis documents that were very diverse in format, content, and quality, this heterogeneity might have affected our thematic analysis. While we are aware of this limitation, we believe that more selective inclusion criteria would have defeated the exploratory purpose of our review. Finally, inductive thematic analyses are also vulnerable to the problem of subjective interpretability by different researchers. This subjective bias is due to the methodological freedom in constructing themes by grouping codes inductively derived from the texts. Although other researchers might have chosen different classification systems, we assessed our thematic classifications iteratively and adapted them along the way to verify their consistency and adherence to the data.

## DISCUSSION

**T**he literature review we conducted illustrates a growing corpus of soft‐law documents on the ethics review of health‐related big data science. The overall number of documents published on this topic increased linearly from year 2012 onwards, indicating a growing interest among regulators and other stakeholders. At the same time, the heterogeneous corpus of documents is indicative of a fragmented ethical and regulatory landscape rather than of an internationally shared framework for ethically aligned big data research. The spectrum of actors involved in this domain is diverse, as it includes, among others, regional (such as the Information and Privacy Commissioner of Ontario), governmental (such as the U. S. Office for Human Research Protections), intergovernmental (such as UNESCO), and supranational (such as the Council of Europe and the European Commission) institutions as well as private companies and NGOs. Very few documents were issued by academic research institutions, despite their direct involvement in research. In contrast, a considerable number of documents were issued by professional associations such as the United Kingdom's Royal Statistical Society and the Internet Association of Privacy Professionals. An even smaller portion of the corpus is represented by independent ethics bodies such as the Nuffield Council on Bioethics and the Italian National Bioethics Committee. However, their documents appeared ethically richer and more detailed compared to the average—an observation that is corroborated by the higher‐than‐average number of codes identified among these documents.

While it cannot be ruled out that private actors’ involvement in big data ethics is indicative of a genuine ethical interest, it has been observed that their proactive guidance efforts have scarce democratic accountability and might raise a risk of undue influence on policy‐making, especially when applied to pervasive systems such as data analytics and artificial intelligence.^38^ In fact, many large health‐related datasets are exclusive property of companies, whose data handling and operational strategies are often hidden by nondisclosure agreements. Given the critical role of private corporations in the data economy, this industry mobilization is necessary to shape an enforceable ethical framework for big data research. At the same time, it raises the quandary of social accountability and the risk that nonstate actors might acquire a quasilegislative power. These problems have particular significance when procedural or substantive conflicts arise between the recommendations provided by, respectively, industry actors and governmental bodies.

Content analysis reveals that most documents provide general normative recommendations about the ethical, legal, and social implications of big data without specifying to which domain these recommendations apply. Moreover, from these documents, it is not clear which actors or bodies should be entitled to promote or enforce these recommendations. Only a minority of documents (33%) specifically addressed IRBs or other ethics review bodies by developing ad hoc recommendations for the review of big data projects. The reason for that is possibly twofold. First, issuer groups such as professional associations, NGOs, and private companies rarely engage with IRBs. Second, big data studies that do not involve human subjects are often perceived as falling outside the purview of ethics review.^39^ This interpretation is corroborated by documents such as the Menlo Report^40^ and the Data & Society Report,^41^ which reveal that researchers involved in data‐intensive research typically avoid formal ethics review, as they do not perceive it to be “human subjects research,” especially when they rely on secondary deidentified data collections or on corporate‐owned databases. This result is consistent with previous studies^42^ showing that researchers using big data methods are more likely to bypass IRB review and to adopt self‐assessment. While self‐regulation approaches are well‐suited to ensure scientific freedom, bypassing ethics review via self‐assessment is ethically problematic. As the history of biomedical ethics has repeatedly shown, the avoidance of independent ethics review can lead to individual or societal harm and diminish the public's trust in science.^43^ This is particularly true for novel areas of science whose ethical boundaries and long‐term consequences are still subject to predictive uncertainty.

Both public and private actors focused their recommendations on defining the conditions for ethically sound acquisition, processing, and storage of data. The remarkable frequency of codes related to privacy and informed consent indicates a prominent ethical and practical concern around these themes. Nevertheless, ethics of big data should not be reduced solely to a privacy issue.^44^ Previous research has observed that, although privacy is a fundamental topic in big data research, it has been overemphasized to the detriment of other issues.^45^ Our findings seem to confirm this observation. Our results also indicate that ethical issues of fairness and data ownership are rarely addressed in current guidance documents. This is concerning given the largely reported risks of bias, discrimination, and informational disenfranchisement associated with algorithms and big data analytics.^46^ Our results are consistent with previous studies about the governance of artificial intelligence (AI) technologies that found interpretative differences and a lack of actionable requirements for the promotion of fairness and justice in the use of these technologies.^47^ These results indicate that attention is missing in this still‐developing area of research ethics, and they attest to persistent uncertainty on how fairness and justice considerations should be addressed in the age of big data and AI. We argue that more detailed normative guidance is needed in this regard.

The high level of interconnectedness among different ethical macrothemes highlights that ethical issues are not in silos but are intimately intertwined. This makes the ethics review of big data projects a complex and multifaceted process that involves not only scrutinizing ethical codes and methods but also inspecting technical requirements, addressing epistemological considerations, and anticipating societal implications. Results suggest that IRBs should exercise their role of essential control systems evaluating and balancing the different faces of each issue, which might require expanded purview and diversified expertise.

Given the fragmentation and heterogeneity of the current landscape of guidance documents, it is unlikely to reduce uncertainty among researchers and IRBs regarding the ethics review of health‐related big data studies. Nonetheless, our results revealed a recurrence of four major procedural recommendations for IRBs. These recommendations address how IRBs should improve their review activities, strengthen their competencies, and revise some of their established practices.

First, documents identified a need for more comprehensive oversight strategies, especially by expanding the purview of IRBs to require formal ethical assessment of data‐intensive studies even when they do not involve the recruitment of human subjects or operate on publicly available data repositories. At the same time, researchers should interpret ethics review not as a waiver of their responsibility but, rather, as an essential quality control of their research. While expanding the purview of IRBs might require new legislation, encouraging researchers to undergo an ethics review on a voluntary basis might be a temporary measure to improve ethical safeguards. Voluntary submissions for review can be incentivized through awareness‐sensitive campaigns about the ethical implications of big data among researchers and by fast‐track review procedures for projects that ensure certain technical requirements.

Second, documents urged IRBs, research institutions, and science regulators to improve the ethics review process and formalize a coherent ethical review framework for the evaluation of big data projects. Documents observed that research ethics paradigms developed for offline research are hardly transferable to data‐driven research in absence of calibration. For example, research aimed at mining health‐related data might have challenging implications for conceptual milestones of human research ethics such as the notion of minimal risk. Unlike conventional research involving human subjects, big data research involving human‐related data might not pose direct risks for the physical integrity of research participants. However, the last few years have borne out the fact that poorly managed datasets can have harmful consequences for human subjects in terms of mental well‐being, harm to reputation, unfair treatment, and discrimination or other forms of informational risk and dignitary harm.^48^ Consequently, the standards of minimal risks developed for clinical research are hardly applicable to the big data domain if significant conceptual and normative adjustments are not performed.

Third, documents highlight the importance of empowering IRBs with the relevant expertise to account for the computational and ethical complexity of big data studies. IRB members trained in medicine, psychology, law, or traditional research ethics might lack the relevant expertise to determine whether, for instance, a certain project is deploying safeguards to avoid algorithmic discrimination, if the machine learning models used for decision‐making are amenable to ex ante and post hoc inspection, or if group‐level privacy risks can arise from the combination of differently structured data sources. Documents suggest that this epistemological gap can be filled with a two‐pronged approach: by diversifying the IRB's composition and through capacity‐building strategies. To diversify their composition, IRBs should consider appointing individuals with expertise in computer science, data analytics, statistics, and data ethics. Furthermore, they should consider the organization of training programs or other educational and capacity‐building activities.

Expanding the IRB purview and their members’ expertise is a requirement grounded on the assumption that IRBs should be the relevant oversight body of big data research. This assumption was not shared unanimously. A few documents addressed the issue of whether IRBs should be the oversight body accountable for big data research at all.^49^ For example, data protection officers were proposed as complementary oversight resources. The creation of novel oversight bodies such as data boards was also proposed as an adaptive governance solution to the big data ethics conundrum.

Finally, documents highlighted the importance of sensitizing researchers and other relevant actors (for instance, technology developers, data analysts, advertisers, insurers, and physicians) about data ethics. The persistent absence of an agreed‐upon ethical framework for big data research might perpetuate uncertainty between both researchers and IRBs and could result in divergent approval decisions. Raising awareness within the research community can help reduce this uncertainty through proactive measures such as the development of codes of ethics and professional conduct (as done by the Association for Computing Machinery and the British Computer Science Association), research roadmaps (as done by the Association of the British Pharmaceutical Industry), or best practices (as done by the Health IT Policy Committee of the United States). Any development of an ethical framework for big data research, however, cannot disregard the active involvement of IRBs in decision‐making. On the contrary, research on the views, needs, and attitudes of IRB members is highly necessary to set an evidence‐based, empirically informed agenda for big data and research ethics.

Despite the prevalence of the above‐listed recurrent themes across documents, there is still much uncertainty about how the recommendations should be implemented. For instance, it is not clear yet how, in practice, IRBs should improve the ethics review process and which recommendation should be implemented first. Whether IRBs should be the bodies devoted to assessing big data projects at all is still debatable. Alternatives might involve universities providing ethical requirements to their researchers who collect, store, or use big data. Additionally, peer‐reviewed journals might set the rule to reject all those publications that do not follow specific ethical procedures and criteria. Another option could involve producing new legislation that includes new research ethics best practices. When advancing this ethical discussion, it is critical that IRBs are not considered passive recipients of guidelines but are actively involved in the norm‐development process. To achieve this aim, qualitative studies assessing the views, needs, and attitudes of IRBs as well as collaborative approaches to guideline development are highly needed.

## Supporting information

The figures and appendices are available in the “Supporting Information” section for the online version of this article and via *Ethics & Human Research*'s “Supporting Information” page: https://www.thehastingscenter.org/supporting-information-ehr/.

Supporting Information
