# Supplementary material for: Big Data, Biomedical Research, and Ethics Review: New Challenges for IRBs
Source: Ethics Hum Res. 2020 Sep 16;42(5):17–28. doi: 10.1002/eahr.500065 (PMC7814666; doi:10.1002/eahr.500065)
Supplement: Supplementary file 1 — Supporting Information [file EAHR-42-17-s001.pdf]

Big Data, Biomedical Research, and Ethics Review: *New Challenges for IRBs*

Agata Ferretti, Marcello Ienca, Samia Hurst, and Effy Vayena

Figure 1.
Distribution of Documents by Documents' Issuers and Targeted Stakeholder Groups

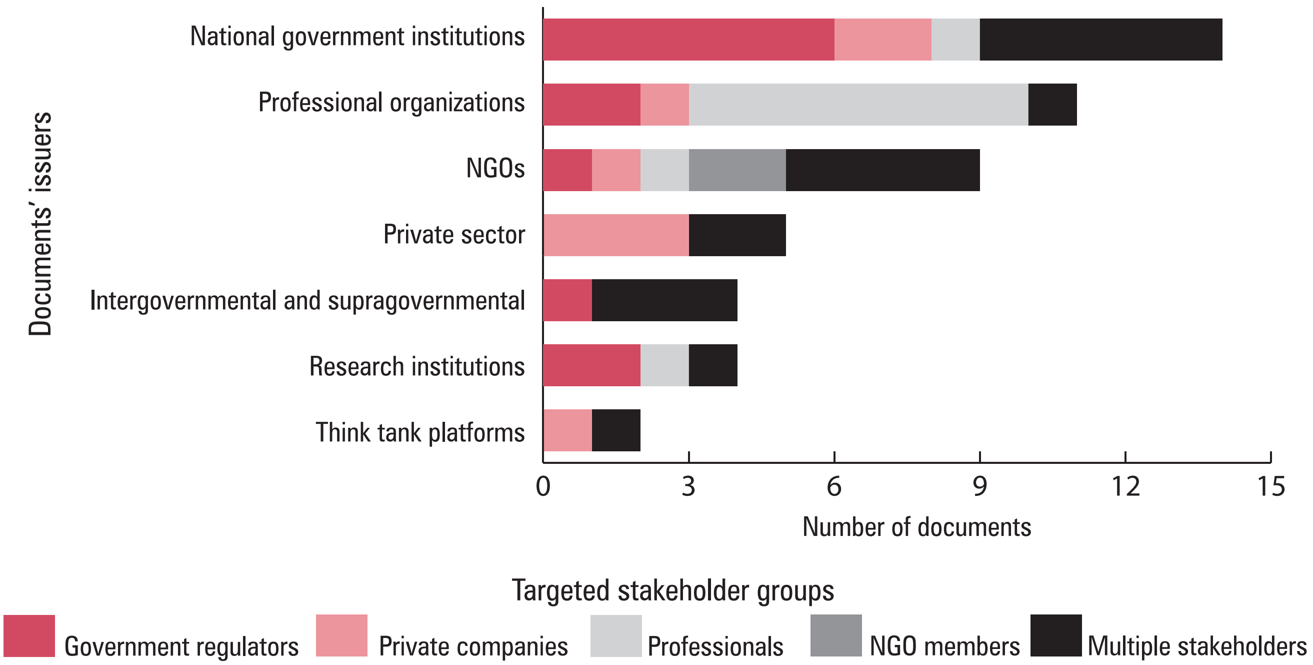

Figure 2.
Alluvial Diagram Showing the Interconnectedness across Multiple Themes and Contextual Thematic Families

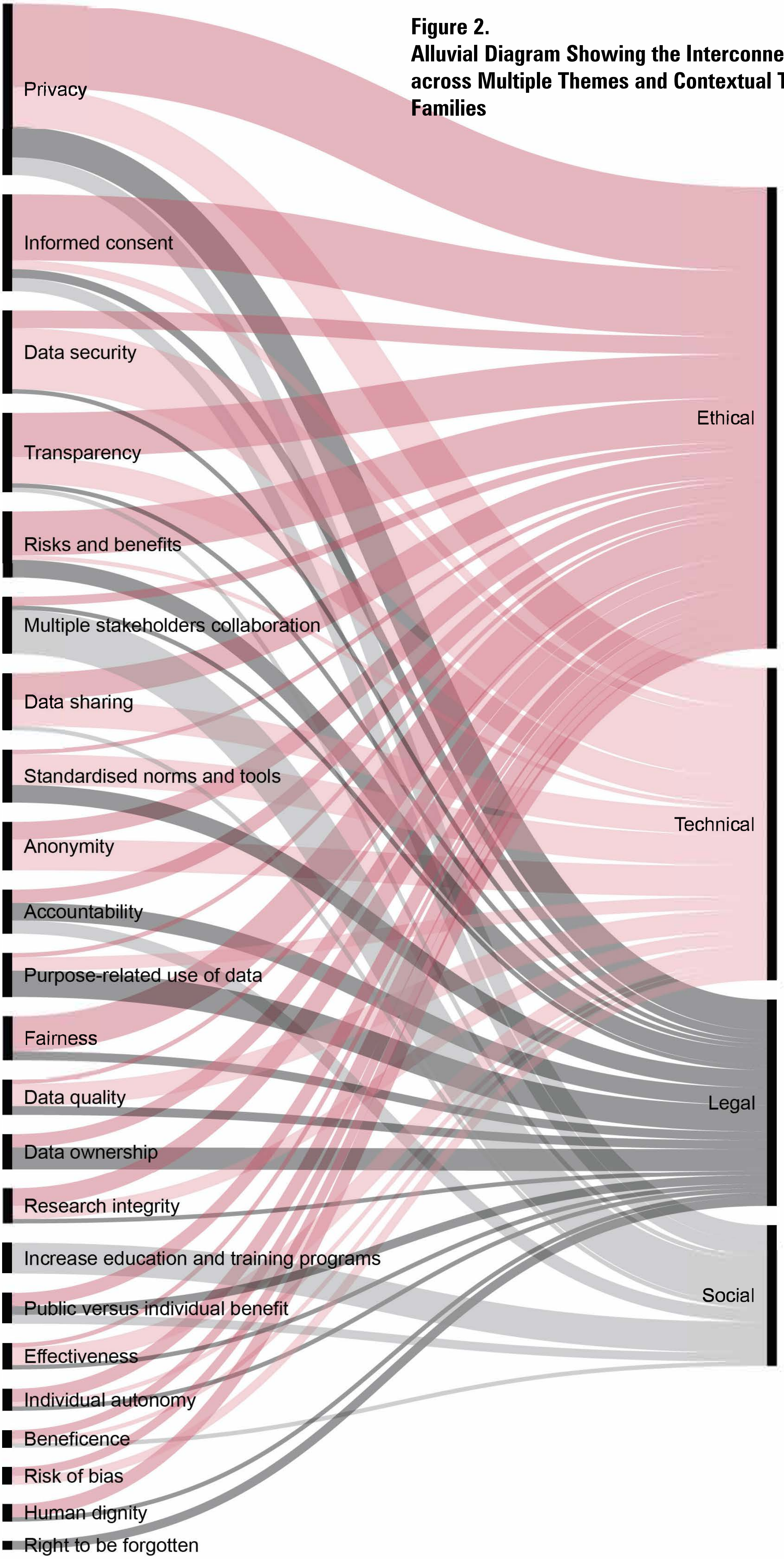

Figure 3.
Substantive Thematic Families of Recommendations in Big Data Research

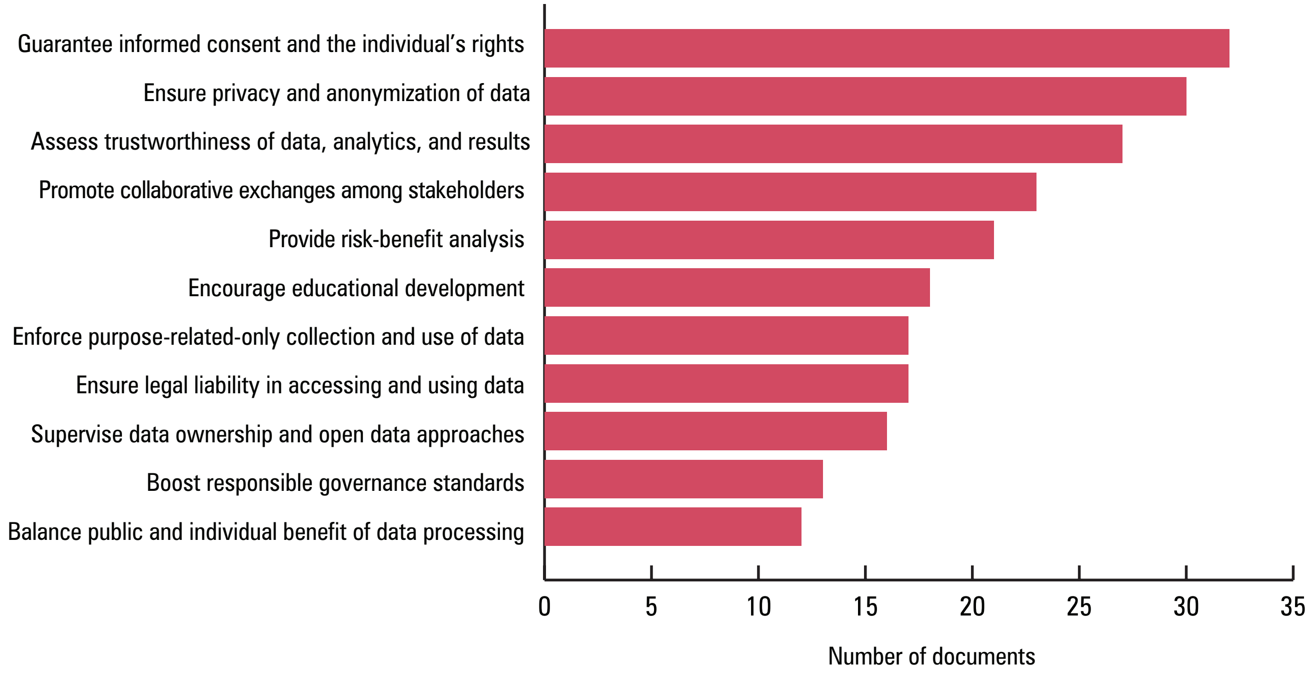

Appendix 1.

List of Included Documents

Title	Issued by	Year	Link	Country of issuer	Type of issuer	Targeted stakeholder group	Recommendations for IRBs	Prominent health focus
Universal Principles of Data Ethics: 12 Guidelines for Developing Ethics Codes	Accenture	2016	https://www.accenture.com/t20160629T012639Z__w__/us-en/_acnmedia/PDF-24/Accenture-Universal-Principles-Data-Ethics.pdf	Ireland	Private sector	Multiple stakeholders	Yes	No
Guiding Principles for the Ethical Use of Data	Acxiom Corporation	2019	https://marketing.acxiom.com/rs/982-LRE-196/images/ACX001_EthicalUseofData.pdf	United States	Private sector	Multiple stakeholders	No	No
National and Transnational Security Implications of Big Data in the Life Sciences	American Association for the Advancement of Science (AAAS)	2014	https://www.aaas.org/sites/default/files/AAAS-FBI-UNICRI_Big_Data_Report_111014.pdf	United States	Professional organization	Government regulators	No	Yes
Ethical Guidelines for Statistical Practice	American Statistical Association	2016	http://www.amstat.org/asa/files/pdfs/EthicalGuidelines.pdf	United States	Professional organization	Professionals	No	Yes
ACM Code of Ethics and Professional Conduct	Association for Computing Machinery	2018	https://www.acm.org/about-acm/acm-code-of-ethics-and-professional-conduct	Multicountry	Professional organization	Professionals	No	No
Ethical Decision-Making and Internet Research 2.0: Recommendations from the AoIR Ethics Working Committee	Association of Internet Researchers, Ethics Working Committee	2012	http://aoir.org/ethics/	Multicountry	Professional organization	Government regulators	Yes	No
Big Data Roadmap	Association of the British Pharmaceutical Industry	2013	http://www.abpi.org.uk/publications/big-data-road-map	United Kingdom	Professional organization	Private companies	No	Yes
Guide to Big Data and the Australian Privacy Principles	Australian Government, Office of the Australian Information Commissioner	2016	https://www.oaic.gov.au/engage-with-us/consultations/guide-to-big-data-and-the-australian-privacy-principles/consultation-draft-guide-to-big-data-and-the-australian-privacy-principles	Australia	National government institution	Multiple stakeholders	No	Yes
Code of Good Practice	British Computer Science Association	2011	http://www.bcs.org/upload/pdf/cop.pdf	United Kingdom	Professional organization	Professionals	No	No
Big Data Roadmap and Cross-Disciplinary Community for Addressing Societal Externalities	BYTE Project	2014	http://new.byte-project.eu/wp-content/uploads/2014/02/D7.3-Final-report-FINAL.pdf	European Union	Intergovernmental and supragovernmental institutions	Multiple stakeholders	No	No
Big Data Analytics in Health: White Paper (Full Report)	Canada Health Infoway	2013	https://www.infoway-inforoute.ca/en/component/edocman/1246-big-data-analytics-in-health-white-paper-full-report/view-document?Itemid=0	Canada	NGO	Multiple stakeholders	No	Yes
Better Information for Improved Health: A Vision for Health System Use of Data in Canada	Canadian Institute for Health Information	2013	https://www.cihi.ca/en/hsu_vision_report_en.pdf	Canada	National government institution	Multiple stakeholders	No	Yes
Health Big Data in the Clinical Context	Center for Democracy and Technology	2015	https://cdt.org/files/2015/04/Health-Big-Data-in-the-Clinical-Context.pdf	United States	NGO	Private companies	No	Yes

**Big Data and Analytics: Seeking Foundations for Effective Privacy Guidance**	Centre for Information Policy Leadership	2013	https://www.hunton.com/files/Uploads/Documents/News_files/Big_Data_and_Analytics_February_2013.pdf	Multicountry	Think tank platform	Private companies	No	Yes
**Big Data Security and Privacy Handbook: 100 Best Practices in Big Data Security and Privacy**	Cloud Security Alliance	2016	https://downloads.cloudsecurityalliance.org/assets/research/big-data/BigData_Security_and_Privacy_Handbook.pdf	Multicountry	NGO	Private companies	No	No
Tecnologie dell'informazione e della comunicazione e big data: Profili bioetici	Presidenza del Consiglio dei Ministri, Comitato Nazionale per la Bioetica	2016	http://www.quotidianosanita.it/allegati/allegato7545104.pdf	Italy	National government institution	Government regulators	No	Yes
**Perspectives on Big Data, Ethics, and Society**	The Council for Big Data, Ethics, and Society	2016	https://bdes.datasociety.net/wp-content/uploads/2016/05/Perspectives-on-Big-Data.pdf	United States	Research institution	Government regulators	Yes	No
**Guidelines on the Protection of Individuals with Regard to the Processing of Personal Data in a World of Big Data**	Council of Europe	2017	https://rm.coe.int/16806ebe7a","https://rm.coe.int/16806ebe7a	European Union	Intergovernmental and supragovernmental institutions	Multiple stakeholders	Yes	No
**Supporting Ethical Data Research: An Exploratory Study of Emerging Issues in Big Data and Technical Research**	Data & Society	2016	https://www.datasociety.net/pubs/sedr/SupportingEthicsDataResearch_Sept2016.pdf	United States	Research institution	Professionals (researchers)	Yes	No
**Data Science Code of Conduct**	Data Science Association	Accessed April 2019	http://www.datascienceassn.org/code-of-conduct.html	United States	Professional organization	Professionals	No	No
**The Menlo Report: Ethical Principles Guiding Information and Communication Technology Research**	Department of Homeland Security, Center for Applied Internet Data Analysis	2012	https://www.caida.org/publications/papers/2012/menlo_report_actual_formatted/menlo_report_actual_formatted.pdf	United States	National government institution	Multiple stakeholders	Yes	No
**Guidance for Incorporating Big Data into Humanitarian Operations**	Digital Humanitarian Network	2015	http://digitalhumanitarians.com/sites/default/files/resource-field_media/IncorporatingBigDataintoHumanitarianOps-2015.pdf	Multicountry	NGO	NGO members	No	No
**The Use of Big Data in Public Health Policy and Research**	European Commission	2014	https://ec.europa.eu/health/sites/health/files/ehealth/docs/ev_20141118_co07b_en.pdf	European Union	Intergovernmental and supragovernmental institutions	Multiple stakeholders	No	Yes
**Big Data in Healthcare—What Role for the EU?**	European Health Parliament	2017	https://www.healthparliament.eu/wp-content/uploads/2017/10/Big-data-in-Healthcare-the-experience-and-results-from-the-European-Health-Parliament.pdf	European Union	Intergovernmental and supragovernmental institutions	Government regulators	No	Yes
**Big Data: A Tool for Inclusion or Exclusion? Understanding the Issues (FTC Report)**	Federal Trade Commission	2016	https://www.ftc.gov/system/files/documents/reports/big-data-tool-inclusion-or-exclusion-understanding-issues/160106big-data-rpt.pdf	United States	National government institution	Private companies	No	No
**Benefit-Risk Analysis for Big Data Projects**	Future of Privacy Forum	2014	https://fpf.org/wp-content/uploads/FPF_DataBenefitAnalysis_FINAL.pdf	United States	Think tank platform	Multiple stakeholders	Yes	Yes
**Health Big Data Recommendations**	Health IT Policy Committee, Health Information Technology Advisory Committee	2015	https://www.healthit.gov/sites/default/files/facas/HITPC_Health_Big_Data_Report_FINAL.pdf	United States	National government institution	Government regulators	Yes	Yes

**Ethics for Big Data and Analytics (white paper)**	IBM	2014	http://www.ibmbigdatahub.com/sites/default/files/whitepapers_reports_file/TCG%20Study%20Report%20-%20Ethics%20for%20BD%26A.pdf	United States	Private sector	Multiple stakeholders	No	No
**Big Data, Artificial Intelligence, Machine Learning and Data Protection**	Information Commissioner's Office	2017	https://ico.org.uk/media/for-organisations/documents/2013559/big-data-ai-ml-and-data-protection.pdf	United Kingdom	National government institution	Private companies	Yes	Yes
**IEEE Code of Ethics**	IEEE	Accessed April 2019	http://www.ieee.org/about/corporate/governance/p7-8.html	Multicountry	Professional organization	Professionals	No	No
**Code of Ethics/Conduct**	INFORM for the Certified Analytics Professional	Accessed April 2019	https://www.certifiedanalytics.org/ethics.php	United States	Professional organization	Professionals	No	No
**Big Data Guidelines**	Information and Privacy Commissioner of Ontario	2017	https://www.ipc.on.ca/wp-content/uploads/2017/05/bigdata-guidelines.pdf	Canada	National government institution	Government regulators	Yes	No
**Building Ethics into Privacy Frameworks for Big Data and AI**	International Association of Privacy Professionals	2018	https://iapp.org/media/pdf/resource_center/BUILDING-ETHICS-INTO-PRIVACY-FRAMEWORKS-FOR-BIG-DATA-AND-AI-UN-Global-Pulse-IAPP.pdf	Multicountry	Professional organization	Multiple stakeholders	Yes	No
**Location Data Privacy: Guidelines, Assessment & Evaluations**	Location Forum	2013	https://iapp.org/media/pdf/resource_center/LocationDataPrivacyGuidelines_v2.pdf	Multicountry	NGO	Multiple stakeholders	No	Yes
Leitlinien für den* Big-Data-*Einsatz im Überblick Chancen und Verantwortung	Nationaler IT-Gipfel	2015	https://www.digitale-technologien.de/DT/Redaktion/DE/Downloads/Publikation/Smart_Data_Positionspapier_Big Data_Leitlinien.pdf?__blob=publicationFile&v=7	Germany	National government institution	Multiple stakeholders	No	Yes
**The Big Data Dilemma**	Nuffield Council on Bioethics	2015	http://nuffieldbioethics.org/wp-content/uploads/Big-Data-dilemma-Nuffield-Council-on-Bioethics-September-2015.pdf	United Kingdom	NGO	Government regulators	No	Yes
**The Collection, Linking and Use of Data in Biomedical Research and Health Care: Ethical Issues**	Nuffield Council on Bioethics	2015	http://nuffieldbioethics.org/wp-content/uploads/Biological_and_health_data_web.pdf	United Kingdom	NGO	Multiple stakeholders	Yes	Yes
**Human Subjects Research Implications of "Big Data" Studies**	Office for Human Research Protections	2015	https://www.hhs.gov/ohrp/sachrp-committee/recommendations/2015-april-24-attachment-a/index.html	United States	National government institution	Government regulators	Yes	Yes
**An Enterprise Architect's Guide to Big Data**	Oracle	2016	http://www.oracle.com/technetwork/topics/entarch/articles/oea-big-data-guide-1522052.pdf	United States	Private sector	Private companies	No	Yes
**The Opportunities and Ethics of Big Data**	Royal Statistical Society	2015	http://www.rss.org.uk/Images/PDF/influencing-change/2016/rss-report-opps-and-ethics-of-big-data-feb-2016.pdf	United Kingdom	Professional organization	Professionals	Yes	No
**The Open Data Era in Health and Social Care**	The GovLab (for NHS England)	2014	http://images.thegovlab.org/wordpress/wp-content/uploads/2014/10/nhs-full-report-21.pdf	United Kingdom	National government institution	Multiple stakeholders	No	Yes

**Big Data, Big Possibilities: How Australia Can Use Big Data for Better Healthcare**	The McKell Institute	2016	https://www.allens.com.au/pubs/pdf/healthcare/Healthcare-McKellReport.pdf	Australia	Research institution	Government regulators	No	Yes
**Big Data and Data Sharing: Ethical Issues**	U.K. Data Service	2017	https://www.ukdataservice.ac.uk/media/604711/big-data-and-data-sharing_ethical-issues.pdf	United Kingdom	National government institution	Professionals	Yes	Yes
**Data Ethics Framework**	U.K. Government, Cabinet Office	2018	https://www.gov.uk/government/publications/data-science-ethical-framework	United Kingdom	National government institution	Government regulators	No	No
**Integrating Big Data into the Monitoring and Evaluation of Development Programmes**	U.N. Global Pulse	2016	http://unglobalpulse.org/sites/default/files/IntegratingBigData_intoMEDP_web_UNGP.pdf	International	NGO	NGO members	No	No
**Draft Report on Big Data and Health**	UNESCO IBC	2017	http://unesdoc.unesco.org/images/0024/002487/248724E.pdf	International	NGO	Multiple stakeholders	Yes	Yes
**The Federal Big Data Research and Development Strategic Plan**	U.S. Subcommittee on Networking and Information Technology Research and Development	2016	https://www.nitrd.gov/PUBS/bigdatardstrategicplan.pdf	United States	National government institution	Government regulators	No	Yes
**Big Data: *Ethische Fragen***	Vodafone Institut für Gesellschaft und Kommunikation	2016	http://www.vodafone-institut.de/wp-content/uploads/2016/10/Big-Data_Ethische-Fragen.pdf	Germany	Private sector	Multiple stakeholders	No	No
**How Should Health Data Be Used? Privacy, Secondary Use, and Big Data Sales**	Yale University, Institution of Social and Policy Studies	2014	http://bioethics.yale.edu/sites/default/files/files/ISPS14-025.pdf	United States	Research institution	Multiple stakeholders	No	Yes

Appendix 2.

Inclusion and Exclusion Criteria for the Literature Review

**Sources considered**	Types	Documents published online or websites featuring materials such as policy documents, soft-law documents, best-practice guidelines, reports, declarations, technical specifications and standards, technical and commercial documentation, and official recommendation documents
	Issuers	National government or international institutions, the private sector (e.g., companies and corporations), NGOs, nonprofit organizations, academic and research institutions, and professional organizations
	Languages	English, Italian, French, German, and Greek (the languages spoken by the authors)
	Content	Documents referring to the ethics of big data *and* providing normative recommendations or best practices
**Sources excluded**	Types	Blog articles, academic articles, letters to the editors, journalistic articles, legislation, books, conference proceedings, dissertations, videos, images, and audio recordings and podcasts
	Issuers	Single authors
	Languages	Others than those mentioned above
	Content	Documents that do not mention any ethical topic related to big data *or* do not provide best practices and recommendations

EHR

Appendix 3.

Substantive Recommendations for IRBs in Relation to the Type of Issuer and the Issuer's Continent

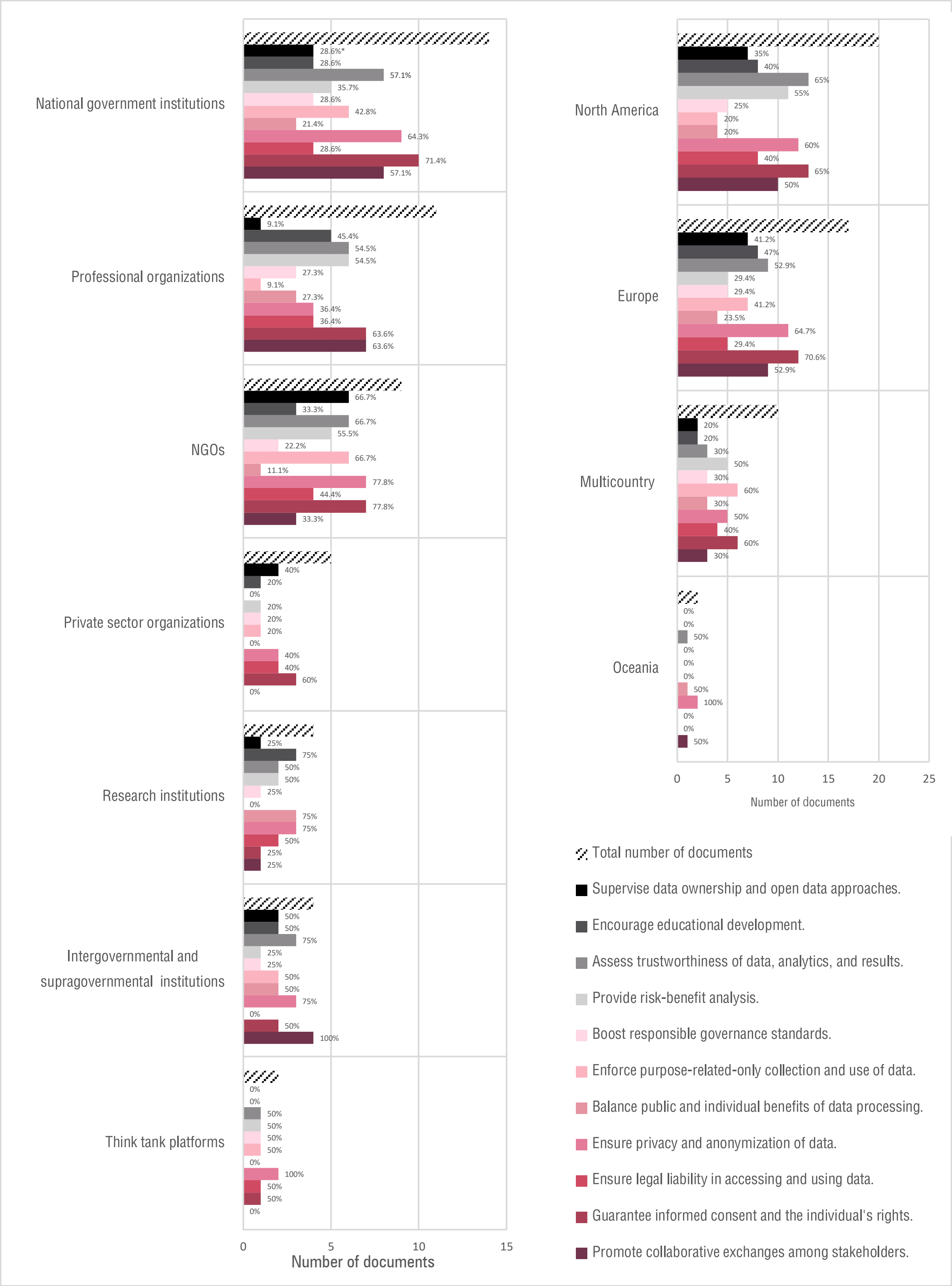

Each percentage refers to the documents within a specific category (e.g., North America or Europe) that mentioned that specific recommendation (e.g., encourage educational development or provide risk-benefit analysis).

*Our analysis identified few peculiarities concerning the substantive ethical recommendations issued (a) in different continents and (b) by various stakeholders. With respect to different continents, almost 60% of North American documents recommended practicing risk-benefit analysis when using big data for research, compared to 30% of European documents. Furthermore, the topic of enforcing purpose-related-only collection and use of data was only partially discussed within European (40%) and North American (20%) documents compared to international documents (60%). The highest proportion of documents including recommendations on ethical educational development for researchers using big data was provided by European documents. When analyzing with respect to stakeholders, intergovernmental and supragovernmental institutions highlighted—more than any other issuer—the importance of collaborative exchange between stakeholders. Our analysis also showed that 80% of research institutions' documents offered recommendations about balancing public and individual benefits of data processing, compared to the 20% of documents issued by national government institutions. Finally, the data breakdown displayed a lack of substantive recommendations by the private sector. For example, the suggestion to assess the trustworthiness of data, the analytical processes, and the results, is discussed by at least 50% of documents released by every other type of stakeholder, but it is not discussed at all in the documents from the private sector.

Appendix 4.
Procedural Recommendations for IRBs in Relation to the Type of Issuer

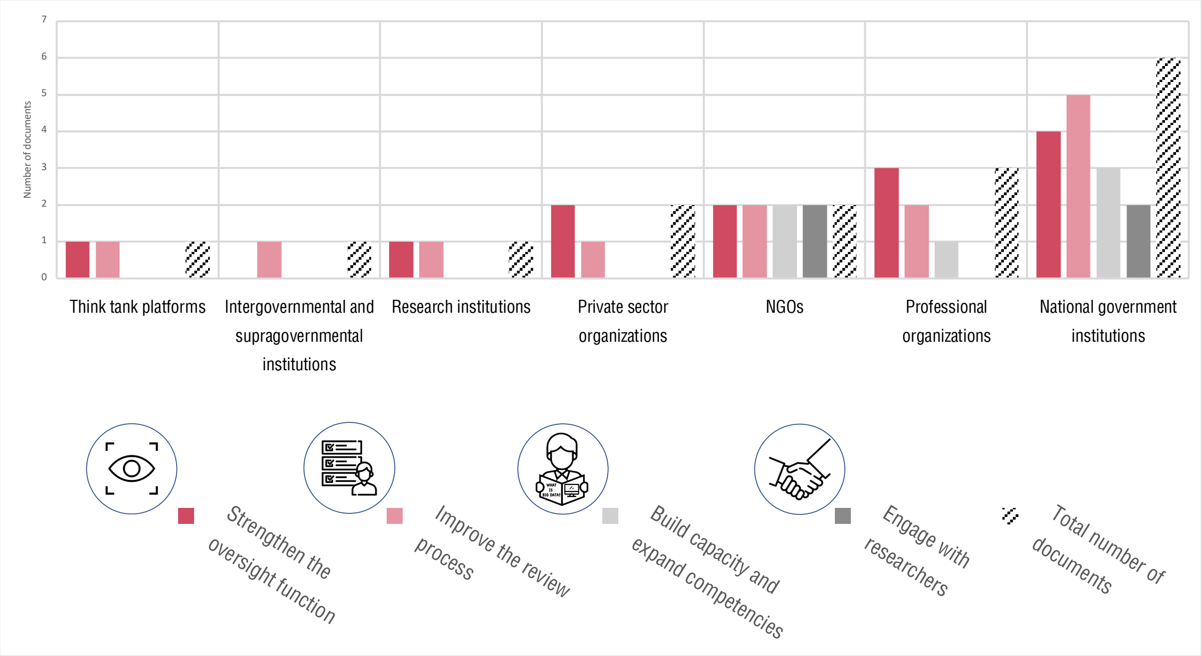

Among all the stakeholders providing procedural recommendations, only national government institutions and research institutions mentioned all four recommendations. In general, there was high heterogeneity across the analyzed documents. For example, none of the documents issued by professional organizations talked about the necessity for IRBs to engage more with researchers, while documents issued by both research institutions and national governments provided this suggestion. Furthermore, the document issued by the private sector focused exclusively on the necessity to strengthen the oversight function of IRBs, while all the other issuers mentioned at least one other recommendation.
